# The Early Impact of Deciding to Take the United States Medical Licensing Examination Step 1 for Osteopathic Medical Students in the Pass/Fail Era

**DOI:** 10.7759/cureus.36154

**Published:** 2023-03-14

**Authors:** Dylan Hedgepeth, Samuel Wlasowicz, Ronald Lott, Travis Smith

**Affiliations:** 1 College of Medicine, Lake Erie College of Osteopathic Medicine, Erie, USA; 2 Clinical Curriculum Integration and Assessment, Lake Erie College of Osteopathic Medicine - Bradenton, Bradenton, USA

**Keywords:** pre-clinical gpa, osteopathic medical education, nbme subject exam, comlex level 1 pass/fail, usmle step 1 pass/fail

## Abstract

Introduction: The United States Medical Licensing Examination (USMLE) Step 1 recently shifted from a three-digit scoring format to a pass/fail scoring rubric. Lake Erie College of Osteopathic Medicine (LECOM) is among a number of osteopathic medical schools that traditionally included passing Step 1 as a graduation requirement. However, LECOM removed this requirement following the change in scoring format. National Board of Medical Examiners (NBME) subject examinations have a significant impact on the clerkship grades of third-year medical students. Therefore, our pilot study compared NBME subject examination scores among third-year LECOM medical students that did and did not take and pass Step 1. We anticipate that both high pre-clinical grade point average (GPA) and having passed Step 1 will be associated with higher subject exam scores, but passing Step 1 will have a relationship with higher subject exam scores that is independent of pre-clinical GPA.

Methods: Using voluntary response sampling, 201 osteopathic medical students from LECOM completed an online survey through Google Forms regarding their pre-clinical GPA, subject exam scores, whether they took and passed USMLE Step 1, and their study resources used throughout clerkships.

Results: There was a positive correlation (*p *< 0.05) found between pre-clinical GPA and exam scores across all subjects among students that took Step 1. There was no relationship between pre-clinical GPA and exam scores across all subjects among students that did not take Step 1 (*p* > 0.05). Students that took Step 1 had a higher pre-clinical GPA than those that did not. Students that took and passed Step 1 scored higher on subject exams. Fifty-nine percent of respondents indicated they would have studied more for Step 1 if these exams were scored on the three-digit format, while zero respondents indicated they would have studied less.

Conclusion: Although higher pre-clinical GPA and taking Step 1 were associated with higher scores on subject exams, taking Step 1 appears to have an independent influence on subject exams because there was no relationship found between pre-clinical GPA and subject exam scores among students that did not take Step 1. Therefore, there may be features related to preparing for this exam that better equip osteopathic medical students to perform well on subject exams.

## Introduction

As of January 26, 2022, the National Board of Medical Examiners (NBME) transitioned the scoring of the United States Medical Licensing Examination (USMLE) Step 1 from a three-digit format to a pass/fail rubric [1]. The National Board of Osteopathic Medical Examiners (NBOME) made the same change on May 10, 2022 for the Comprehensive Osteopathic Medical Licensure Examination (COMLEX) Level 1 [2]. Prior to these changes, medical students’ scores on Step 1 and Level 1 had an important impact on competitiveness for post-graduate year (PGY) residency [3]. In the 2020 National Resident Matching Program (NRMP) Director Survey, 90% of program directors cited Step 1 scores as a vital factor in application review, rating it 4.0/5.0 in importance for evaluating candidates, while Level 1 scores were cited by 56% of residency programs as vital [3]. Therefore, the consequences of losing these exam scores as measurable impact factors for osteopathic medical students warrant further investigation. 

Historically, preparation by students for Step 1 and Level 1 involved the use of a variety of resources, including question banks, NBME Comprehensive Basic Science Exams (CBSE), and third-party video resources [4]. It is reasonable to imagine that having access to a multitude of resources might be overwhelming for students. The correlation between additional resources and performance on Step 1 has been examined previously [5]. For example, Step 1 scores have been found to correlate directly with the percentage of questions answered correctly on UWorld and with NBME CBSE scores [5]. Higher scores on Step 1 have also demonstrated predictive value for high scores on NBME subject exams, with one study showing the use of Step 1 scores to predict subject exam scores within 0.2 points [6]. This relationship between Step 1 scores and subject exam scores was useful, however the recent shift to a pass/fail rubric means this needs to be reexamined. 

Previous research has demonstrated the predictive value of Level 1 scores for Step 1 scores [7]. However, due to concern over the reliability of this predictive value, and due to some residency programs’ unfamiliarity with how Level 1 is scored, osteopathic medical students that do not take Step 1 might be less competitive for residency [8]. Additionally, there is no published NRMP program director data regarding the impact of osteopathic medical students’ choice to take Step 1 on their candidacy for residency. Therefore, the impact of changing Level 1 and Step 1 to a pass/fail rubric is unknown, but assessing NBME subject exam performance among the classes of 2024 may provide valuable insight. The primary purpose of our pilot study was to investigate whether or not osteopathic medical students’ choices to sit for Step 1 had an impact on their subject exam performance.

## Materials and methods

On October 4, 2022, the authors distributed a survey to 649 third-year medical students across all Lake Erie College of Osteopathic Medicine (LECOM) campuses. Two hundred and one (31%) of students voluntarily responded to the survey. The survey was distributed via a secured Outlook e-mail from the Office of Clinical Education at LECOM, and participation was voluntary. Students were offered a second opportunity to complete the survey via e-mail reminder on October 12, 2022. Students were reminded once via email. The survey and data collection continued until November 14, 2022, after which the survey closed. The survey was administered using Google Forms. To maintain anonymity, the ‘collect email addresses’ and ‘limit to one response’ features were unchecked, confirming that respondents did not need to log into their google account to fill out the survey. This study was exempt from Institutional Review Board (IRB) review and approval. For access to specific questions asked in the survey, refer to the following link: https://forms.gle/7wb97C5a3hdGbomU6. A PDF version of the survey is also made available upon request to the corresponding author.

Survey data were downloaded to and organized in Microsoft Excel (Redmond, WA, USA). Upon download, categorical information was tabulated into “dummy” variables (ones and zeros). Incomplete responses were omitted prior to importing the data into RStudio for analyses. A Shapiro-Wilk test confirmed the data were not normally distributed. Mann-Whitney U tests were performed to compare exam scores for each subject across categorical variables. Correlations were computed as Pearson’s coefficients. For all analyses an ⍺ of 0.05 was used to determine significance. 

## Results

**Figure 1:**
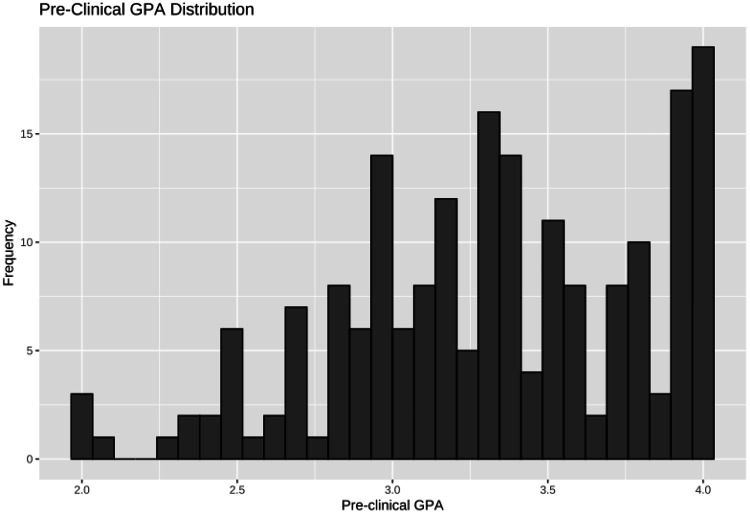
Pre-clinical GPA Frequency Distribution Pre-clinical GPA data among the survey sample. The frequencies are displayed on the y-axis. GPA: Grade Point Average

The distribution for pre-clinical GPA was negatively (left) skewed, a graphical demonstration of which is presented in Figure 1. Numerical value of skewness calculated to 0.062. Shapiro-Wilk test for normality yielded p < 0.001 for respondents’ pre-clinical GPA, warranting use of non-parametric tests for analyses. Median pre-clinical GPA was 3.31. Inter-quartile range (IQR) was 0.75.

With non-parametric testing, median pre-clinical GPA was found to be higher for students that chose to take Step 1 across all subject exams (Table 1). Associated p-values from the Mann-Whitney U tests performed for each subject were statistically significant.

**Table 1:** Mann-Whitney U Tests Comparing Median Pre-clinical GPA for Students Who Did and Did Not Take Step 1 GPA: Grade Point Average

Pre-clinical GPA					
	Did not take Step 1		Took Step 1		
Subject	n	Median	n	Median	p-value
Family Medicine	16	2.8	46	3.6	< 0.001
Internal Medicine	10	2.9	22	3.8	< 0.001
Pediatrics	18	3.0	30	3.6	< 0.001
Surgery	15	2.9	31	3.6	< 0.001
OBGYN	20	3.0	30	3.7	< 0.001
Psychiatry	21	3.0	38	3.5	< 0.001

**Figure 2:**
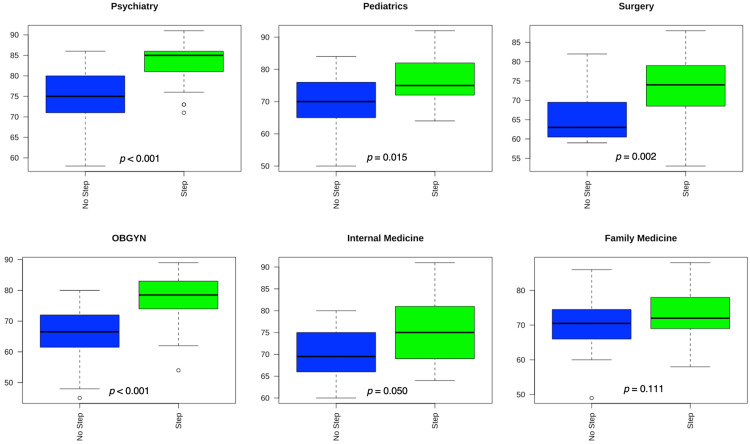
Mann-Whitney U Tests Comparing Subject Exam Scores Between Students Who Did and Did Not Take or Pass USMLE Step 1. Boxplots showing comparisons of subject exam scores. Y-axes indicate subject exam scores. USMLE: United States Medical Licensing Examination

**Figure 3:**
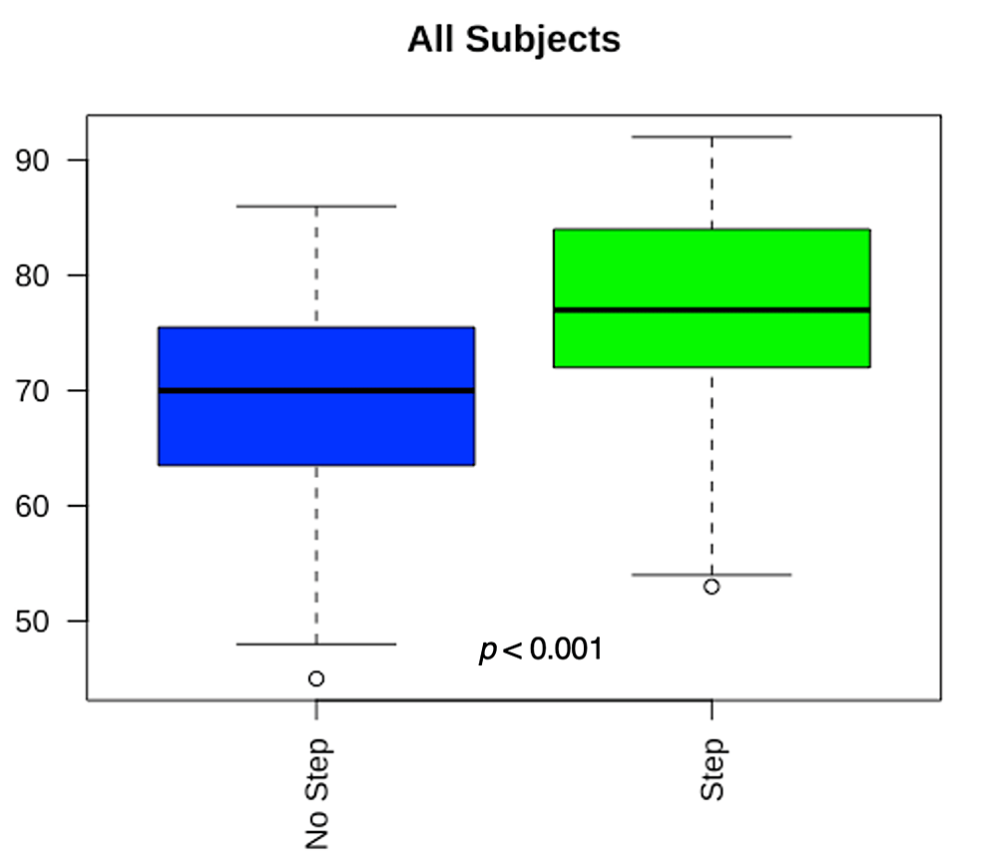
Mann-Whitney U Tests Comparing Subject Exam Scores Across All Subjects Between Students Who Did and Did Not Take or Pass USMLE Step 1. Boxplots showing comparisons of all subject exam scores. Y-axis indicates subject exam scores. USMLE: United States Medical Licensing Examination

Figure 2 illustrates comparison of exam scores for each subject between students that did and did not take Step 1 using Mann Whitney U tests. Figure 3 demonstrates this for exam scores among all subjects. Median exam scores were higher (p < 0.05) among students that took Step 1 for all subjects except for Internal Medicine (p = 0.05) and Family Medicine (p = 0.11). Students who took Step 1 scored higher when including all subjects together (p < 0.001). Y-axes in the following boxplots are numeric and denote subject exam scores. For Figures 2 and 3, ‘No Step’ = Passed Comprehensive Osteopathic Medical Licensing Examination of the United States (COMLEX) Level 1, while ‘Step’ = Passed COMLEX Level 1 and USMLE Step 1. For Figure 2, Psychiatry, n = 23 (No Step) and n = 39 (Step); Internal Medicine, n = 9 (No Step) and n = 24 (Step); Family Medicine, n = 16 (No Step) and n = 46 (Step); Surgery, n = 15 (No Step) and n = 31 (Step); Pediatrics, n = 19 (No Step) and n = 30 (Step); OBGYN, n = 21 (No Step) and n = 30 (Step). For Figure 3, n = 100 (No Step) and n = 197 (Step).

At this point, we found that students who passed both Level 1 and Step 1 had higher pre-clinical GPAs than those that passed Level 1 alone. We also found that students who passed Step 1 scored higher on most subject exams compared to students who did not pass Step 1. The logical remaining question concerns whether or not higher pre-clinical GPA is associated with higher subject exam scores independent of Step 1, or are both factors associated with higher subject exam scores?

We performed correlation analyses to investigate associations between pre-clinical GPA and subject exam scores among students that passed Level 1 alone, among students that passed both Level 1 and Step 1, and among all of these students (“Step 1 agnostic”). Pre-clinical GPA was not positively or negatively correlated with exam scores across all subjects among students that passed Level 1 alone. Among students that passed both Level 1 and Step 1, pre-clinical GPA was positively correlated with exam scores in all subjects except for Internal Medicine and Surgery. Additionally, pre-clinical GPA was positively correlated with exam scores in the Step 1 agnostic group among all subjects except for Internal Medicine (Table 2).

**Table 2:** Correlation Analysis Comparing Pre-clinical GPA Versus Subject Exam Score Pearson coefficients denoted by rho. Significant coefficients marked with an asterisk. ‘Step 1 Agnostic’ includes a cohort of students that both passed Level 1 alone in addition to those who passed both Level 1 and Step 1. GPA: Grade Point Average, NBME: National Board of Medical Examiners

Pre-Clinical GPA vs. NBME Subject Exam Score						
	Did not take Step 1		Took Step 1		Step 1 Agnostic	
Subject	⍴	p-value	⍴	p-value	⍴	p-value
Family Medicine	0.335	0.205	0.339*	0.021	0.398*	0.001
Internal Medicine	-0.351	0.32	0.348	0.112	0.319	0.075
Pediatrics	0.175	0.486	0.687*	<0.001	0.580*	< 0.001
Surgery	0.048	0.864	0.180	0.333	0.378*	0.010
OBGYN	0.434	0.056	0.637*	<0.001	0.694*	< 0.001
Psychiatry	-0.063	0.787	0.378*	0.019	0.447*	< 0.001

## Discussion

NBME subject examinations are an important assessment tool for third-year medical students. Although residency programs may not have access to a student’s subject exam scores, they are factored into the student’s final grades and averaged with other coursework, as well as their preceptor evaluations [9]. Additionally, subject exam scores have demonstrated high correlation with USMLE Step 2 clinical knowledge (CK) scores [10]. Since subject exam scores contribute enough to a student’s grade such that a failure can significantly impact the corresponding clerkship grade and because performance on them correlates with performance on the licensing exam Step 2 CK, scores on subject exams have an important role in students’ candidacies for residency programs.

The results of the correlation analyses performed in this pilot study suggest value in replicating similar analyses on a larger data set in order to better understand the role of Step 1 in osteopathic medical education. Passing both Level 1 and Step 1 (versus passing Level 1 alone) was associated with higher subject exam scores; and higher pre-clinical GPA was found to be associated with passing both Level 1 and Step 1. Therefore, an appropriate next question is: what really drives higher scores on subject exams? Pre-clinical GPA, passing Level 1 and Step 1, or both? Because there was no relationship identified between pre-clinical GPA and subject exam scores among students that did not take Step 1, we cannot definitively suggest that pre-clinical GPA is independently correlated with subject exam performance. Additionally, despite 59% of respondents indicating they would have studied more for Level 1 and/or Step 1 if scored with a three-digit format, taking Step 1 was nonetheless associated with higher subject exam scores. Therefore, we propose that higher pre-clinical GPA and passing Step 1 might jointly assist students in their preparation for subject exams.

Research cited earlier in this article supports that scores on Step 1 could reasonably predict subject exam scores. As we found that students with high pre-clincial GPA were more likely to take Step 1, and that students who took Step 1 scored higher on subject exams, we were unable to isolate the influence of taking Step 1 on subject exam performance. However, because there was no relationship identified between pre-clinical GPA and subject exam scores among students that did not take Step 1, we were able to somewhat isolate the influence of (or lack thereof) pre-clinical GPA on subject exam scores. Although Step 1 is no longer scored with a three-digit rubric, we argue against discounting the preparation for Step 1 as a valuable tool to strengthen rising third-year osteopathic medical students for their clerkships. We caution readers to avoid deferring to aptitude as an underlying explanation of our findings, particularly because students that did not take or pass Step 1 had a similar range of pre-clinical GPAs compared to those that did. 

There are limitations to our pilot study. As the survey was distributed partway through participants’ clerkships, no single student had completed each NBME subject exam. Therefore, although we had 201 respondents (each with one or more subject exam scores reported), sample sizes for comparing two independent variables for each subject were relatively small. We also cannot rule out the influence of response bias, as an abundance of students with higher reported pre-clinical GPA responded to our survey. Finally, although this study investigated exam preparation-related features (e.g., the use of additional resources), it would be beneficial to acquire more detailed information regarding study strategies.

## Conclusions

Optimizing medical education is complex and traditionally relies heavily on quantitative measures of student performance. However, the pass/fail era of USMLE Step 1 and COMLEX Level 1 eliminates valuable measures of such. There are many hypotheses regarding the impact of the pass/fail era on osteopathic medical students’ candidacies for residency, but since there is insufficient data on the matter, further exploration is warranted. The pass/fail era for Step 1 began in January 2022, so NBME subject exams among the classes of 2024 provide the most immediate quantitative assessment of its impact.

While our analyses demonstrated an overall positive relationship between pre-clinical GPA and scores on NBME subject exams; a relationship between higher pre-clinical GPA and choosing to take Step 1; and a relationship between taking Step 1 and higher subject exam scores; there was no relationship between pre-clinical GPA and subject exam scores among students that did not take Step 1. This helps justify the value of further investigation into the possible benefits for osteopathic medical students that undertake the preparation for Step 1. Osteopathic medical students with higher baseline academic performance may be more likely to take Step 1. However, our analyses suggest the possibility of additional, independent value in taking Step 1 with regard to preparation for subject exams during students' third year. Subject exam scores impact clerkship grades, and clerkship grades impact students’ competitiveness for residency. Therefore, more research on the role of Step 1 in the education of osteopathic medical students is warranted so that osteopathic medical schools are adequately informed on the role of Step 1 in the education of their students.
